# Real-Time Rail Electrification Systems Monitoring: A Review of Technologies

**DOI:** 10.3390/s25216625

**Published:** 2025-10-28

**Authors:** Jose A. Sainz-Aja, João Pombo, Jordan Brant, Pedro Antunes, José M. Rebelo, José Santos, Diego Ferreño

**Affiliations:** 1 LADICIM (Laboratory of Science and Engineering of Materials), University of Cantabria, 39005 Santander, Spain; jose.sainz-aja@unican.es (J.A.S.-A.); diego.ferreno@unican.es (D.F.); 2 Institute of Railway Research, University of Huddersfield, Huddersfield HD1 3DH, UK; j.brant2@hud.ac.uk (J.B.); p.antunes@hud.ac.uk (P.A.); j.rebelo@hud.ac.uk (J.M.R.);; 3 IDMEC, Instituto Superior Técnico, Universidade de Lisboa, 1049-001 Lisboa, Portugal; 4 ISEL, IPL, 1959-007 Lisboa, Portugal

**Keywords:** pantograph–overhead contact line interface, condition monitoring, railway maintenance strategies, decision support, service safety and reliability

## Abstract

**Highlights:**

**What are the main findings?**

- Identify the current limitations of pantograph–catenary monitoring systems;
- Realise the industry’s needs in terms of monitoring systems for rail electrification assets;
- Real-time knowledge of current collection performance during train service.

**What are the implications of the main findings?**

- Open perspectives for the development of novel technologies to monitor current collection performance;
- Reduce the frequency of maintenance interventions and extend the life-cycle of railway assets.

**Abstract:**

Most electrified railway networks are powered through a pantograph–overhead contact line (OCL) interface to ensure safe and reliable operation. The OCL is one of the most vulnerable components of the train traction power system as it is subjected to multiple impacts from the pantographs and to unpredictable environmental conditions. Wear, mounting imperfections, contact incidents, weather conditions, and inadequate maintenance lead to increased degradation of the pantograph–OCL current collection performance, causing degradation on contacting elements and assets failure. Incidents involving the pantograph–OCL system are significant sources of traffic disruption and train delays, e.g., Network Rail statistics show that, on average, delays due to OCL failures are 2500 h per year. In recent years, maintenance strategies have evolved significantly with improvements in technology and the increased interest in using real-time and historical data in decision support. This has led to an expansion in sensing systems for structures, vehicles, and machinery. The railway industry is currently investing in condition monitoring (CM) technologies in order to achieve lower failure rates and increase the availability, reliability, and safety of the railway service. This work presents a comprehensive review of the current CM systems for the pantograph–OCL, including their advantages and disadvantages, and outlines future trends in this area.

## 1. Introduction

Demand for global passenger and freight transport is anticipated to more than double by 2050 [1,2], underlining the urgency for deep transformations in transportation systems to address societal demands and global environmental challenges. Within this scenario, railways emerge as a highly efficient channel, yielding benefits in both passenger and freight transport sectors [3,4]. Railways represent 2% of total transport energy consumption, handling 8% of the world’s passengers and 7% of global freight [5]. This illustrates its inherent energy efficiency and potential for CO_2_ emission reduction. The smooth operation of railways hinges greatly on the pantograph–catenary system, a vital yet fragile component that necessitates adequate design, construction, monitoring, and maintenance to ensure its cost-efficiency and safety [6,7,8,9]. Even though the railway’s contribution to greenhouse gas emissions is just 1%, its integral role in the global economy and climate change mitigation is unquestionable. This study seeks to explore and highlight the pivotal role of railways in society and their potential to drive a more efficient energy transition in the transport sector.

**Figure 1:**
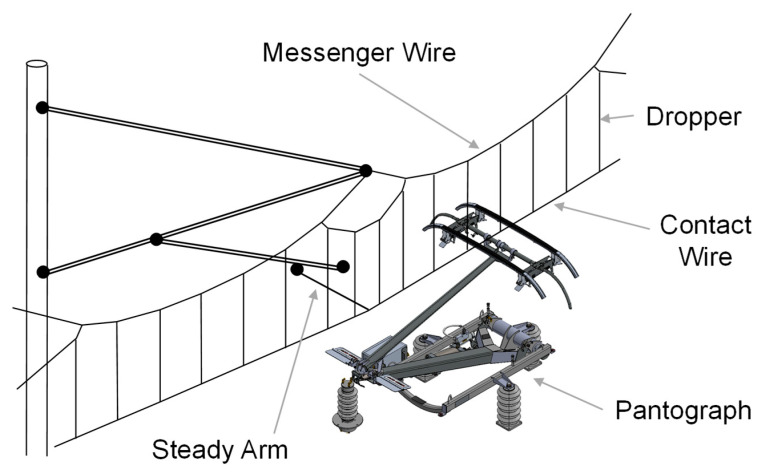
Representation of pantograph–catenary interaction.

Most electrified railway networks draw their power through the pantograph–OCL interface [8,10]. Often, this represents the sole power source for trains, and it is indispensable to maintain the regular operation of the network. In this context, the pantograph and the catenary, depicted in Figure 1, become central elements of the electric traction system. The pantograph is a mechanical apparatus situated above trains, which enables the transmission of electrical energy from the OCL, or catenary, to the locomotive. Its particular design helps in maintaining continuous and efficient contact with the OCL, irrespective of variations in train speed, track contour, and environmental conditions [11,12,13,14].

The catenary comprises the system of aerial cables supplying electric current. The term ‘catenary’ originates from the distinct curvilinear form the cables assume when suspended under their own weight, a shape mathematically described as a catenary curve. This specific design allows the cables to resist tensile forces while ensuring a steady power supply [15,16,17]. Thus, an effective interaction between the pantograph and the catenary is crucial for the seamless functioning of the railway’s electric traction system [18,19]. Any disruption or irregularity within this interface could have a detrimental impact on the efficiency and safety of the overall railway transport [20,21,22]. Recent advances in this area allowed the development of reliable computational tools that can study with detail the pantograph–OCL interaction dynamics [23,24,25,26,27,28]. Hence, there is now a larger consensus within the scientific community and industry on the reliability and benefits of these tools.

Despite the above-mentioned developments, global warming effects and extreme weather events are exacerbating the service conditions, even for conventional railway lines. So, in recent years, issues have been reported regarding abnormal wear of the pantograph–catenary interface [29,30,31,32], crosswinds [33,34,35], sandstorms, and thunderstorms [36]. The importance of the health of the current collection assets for the reliability of train operations has driven the development of sensing systems installed on-board trains and wayside in the infrastructure to monitor the pantograph–catenary interaction. The data collected by these systems can then be used for diagnostics, avoiding faults in service, and prognostics, promoting the shift from corrective/preventive maintenance to predictive maintenance [37,38,39,40,41]. Furthermore, the use of digital twins, machine learning techniques, and artificial intelligence-based methods for the health monitoring of railway assets is rapidly expanding [42,43,44].

Pantograph–OCL incidents are significant sources of traffic disruption and train delays. Statistics collected by the Office of Rail and Road in the UK from 2019 to 2021 [45] detail the train delay information due to OCL incidents and are presented in Table 1. There has been a series of high-profile incidents involving the OCL, sometimes exacerbated by unusually hot weather. Some of the incidents were not necessarily very serious in terms of damage to the infrastructure; however, since these incidents make a significant contribution to overall train performance, the disruption consequences were extensive, with hundreds of trains delayed and thousands of delay minutes being accumulated. Pantograph–OCL incidents can also cause fatal accidents and/or threaten the safety of passengers and staff. The accidents that cause severe consequences are generally developed from faults that go undetected. If these faults can be accurately identified and eliminated, the pantograph–OCL performance can be maintained efficiently, reducing the occurrence of incidents, with substantial savings in operation and maintenance costs.

Currently, asset condition monitoring techniques are generally performed by manual inspection using trained personnel; however, manual inspection is lengthy, laborious, and hazardous, and the results are dependent on the capability of the observer to catch possible anomalies and recognise critical situations. Thus, it is important to conduct more effective maintenance and efficient inspection, automating tasks where possible. If the rail industry is to avoid unplanned downtime and continue to meet the growing demands of railway service efficiency, the implementation of CM systems is imperative. This paper summarises state-of-the-art condition monitoring of the pantograph–OCL system, details the existing technologies and representative products, presents their advantages and capabilities, and discusses perspectives of future trends.

This paper is organised as follows: Section 2 introduces the rail industry requirements for monitoring systems and summarises the key parameters that should be measured for CM and fault diagnosis. Section 3 reviews the data acquisition systems available. The key conclusions and future trends are given in Section 4.

## 2. Rail Industry Requirements for Monitoring Systems

Monitoring systems have become a pivotal component of the rail industry, significantly enhancing the operational efficiency, safety, and reliability of services. Various entities within the industry have developed their specific requirements for these systems, with each requiring slightly different criteria that reflect the unique operational contexts and objectives of each entity. The following sections of this review will outline the specific requirements of key stakeholders. These include the European Standards, Schunk customer surveys, Network Rail, ProRail, and the China Railway Corporation (CRC). This analysis will provide an in-depth understanding of the needs and priorities of different organisations within the rail industry. Such insights offer valuable guidance for manufacturers and developers in their pursuit of designing and implementing effective monitoring systems that fulfil the varied and specific requirements of the industry.

- Before detailing the criteria set by key stakeholders, it is important to identify the parameters that can be measured by pantograph–OCL monitoring systems. The physical quantities measurable by these systems vary depending on the capabilities of the sensing devices and the assets to be analysed. These parameters include the following:
- Contact Forces: Forces resulting from pantograph–catenary interaction dynamics.
- Contact Wire Uplift/Height Exceedances: Uplift of the OCL wires caused by the pantograph(s) passage.
- Arcing/Contact Loss: Contact loss events and electric arcing.
- Carbon Strip Wear: Pantograph strip conditions, namely strip wear, chipping, and cracking.
- OCL Hard Spots/Accelerations on Pantograph Head: Pantograph head dynamics caused by OCL singularities and discrete features.
- Contact Position/Wire Stagger/Dewirement: Lateral position of the contact point on the contact strips to obtain the dynamic stagger and evaluate the risk of dewirement.
- OCL/Pantograph Geometry: Catenary and pantograph geometric parameters.
- Dropper/Clamp/Component Defects: Defective critical OCL components and support devices, e.g., loosened, cracked, fractured, broken, or missing components such as the dropper, steady arm, insulator, claw, clevis, double tube joint, two diagonal tubes, pin, and cantilever.
- Worn Contact Wires: Wear state of catenary contact wire (thickness).
- Wire Tension: Tension of the contact and messenger wires.
- Temperature: Temperature of the contact and messenger wires.
- Reporting to Train: Reporting of the measurement data to the train.
- Accident Investigation/Pantograph–Catenary Imagery: Pantograph–catenary imagery to allow accident investigation.
- Trash in OCL: Foreign objects on the catenary, including bird nests, plastic bags, and rocks clipped in the insulators.

### 2.1. European Standard’s Requirements

European Standard EN50367:2020 [19] specifies requirements for the interaction between pantographs and the OCL to achieve free access authorisation to the lines in the European railway network. Table 2 outlines the requirements for gaining free access. Thus, the industry would significantly benefit from monitoring systems capable of precisely measuring these parameters. Notice that, in this table, v is the train velocity in km/h.

### 2.2. Schunk Customer Survey

Pantograph manufacturer Schunk carried out comprehensive market research to understand the requirements of their customers in terms of pantograph–catenary measurements. This market survey considers a diverse customer base from across six European countries, namely Germany, the Netherlands, Spain, Belgium, Sweden, and France.

**Figure 2:**
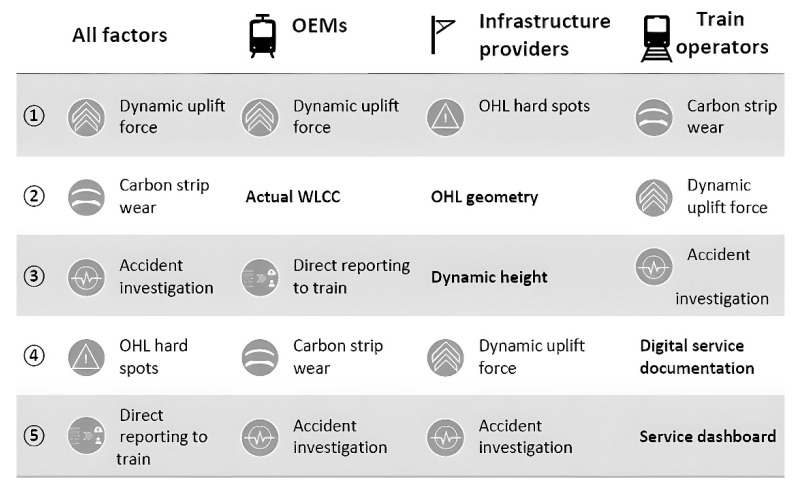
Schunk survey: top five measuring functions for each customer group.

An analysis by the monitoring function approach was taken, giving each function a unique customer score. Schunk provided customers 23 functions for evaluation in two categories, namely direct monitoring and services, and advanced insight. Each function has an average score across original equipment manufacturers (OEMs), infrastructure managers, and train operators, and an individual score per sector. Figure 2 shows the top five customer requirements for each customer group, where OHL means overhead line and WLCC refers to whole life-cycle costs. In bold are the measuring functions that are only important to a certain customer group.

The analysis carried out in the different customer groups shows that, overall, the most consensual physical parameter to measure is the dynamic uplift force. This quantity is of key interest to the rail industry in general as it can determine the free access authorisation for a train fleet to operate in a given network. It is also noticeable that OCL hard spots are the main measurement focus for infrastructure managers. On the other hand, the train operators are mainly concerned about carbon strip wear monitoring.

### 2.3. Network Rail’s Requirements (Requirements in the UK)

As the infrastructure manager in the UK, Network Rail, particularly their OCL monitoring experts, have specified key requirements for pantograph–catenary monitoring capabilities. These are outlined as follows:

- Peak Contact Forces: This is crucial for preventing damage to OCL components, such as neutral sections, section insulators, and crossovers.
- Excessive Wire Stagger: Monitoring this can help prevent horn running and, ultimately, avoid dewirements.
- Longitudinal Acceleration: Monitoring can help prevent carbon chipping or the activation of the Automatic Dropping Device (ADD).
- Wire Height Exceedances (high and low): This monitoring focuses on safety aspects of the OCL.
- Wire Wear (including side wear): Particularly useful when there are converging/diverging wires—it can indicate incorrect setup.
- Pantograph Imagery: This assists in verifying faults and pinpointing where and when they occurred.
- Ability to Detect Defective Components: This enables the detection of detached droppers, incorrectly set up neutral sections, section insulators, and registration arms. Additionally, it can detect anything encroaching into the pantograph envelope, such as vegetation and trash in the OCL.

### 2.4. ProRail’s Requirements (Requirements in the Netherlands)

The following information details the monitoring systems requirements set out by ProRail, the infrastructure manager in the Netherlands. The requirements focus on detecting irregularities in the catenary system, as well as measuring the contact wire and pantograph. ProRail’s requirements for the catenary system include the following:

- Broken Droppers: Broken droppers can lead to the contact wire sagging, which in turn can accelerate its wear.
- Loose Dropper-Contact Wire Clamps: Loose clamps can result in slackened droppers or droppers sagging below the contact wire, causing damage to the pantograph and, ultimately, to the contact wire.
- Sagged Electrical Connections: Electrical connections between the contact wire and messenger wire, if incorrectly mounted or sagged over time, can skew the position of the contact wire clamp. This can cause uneven wear if both contact wires are not passed over equally. It is worth noting that two parallel contact wires are used for AC-100 catenaries in the Netherlands.
- Non-Equally Worn-Down Contact Wires: When the two contact wires are not parallel or at the same height, this can cause uneven wear among the two contact wires. Often, the cause is a skewed clamp position.
- Garbage in the Catenary System: Objects such as plastic bags or deceased birds can cause damage to the catenary system or become entangled in the pantograph, damaging it.

The requirements for the contact wire and pantograph monitoring system deemed critical by ProRail are outlined below:

- Too-Thin Contact Wire: This can lead to contact wire rupture. The system must alarm on the detection of contact wire thickness less than 7.5 mm and must report the contact wire thickness at least every 25 cm.
- Hard Spots: These cause extra contact forces between the pantograph and the contact wire, causing extra wear of the contact wire just before and after these points.
- Parallel Incoming Contact Wire Hanging Too Low: If this occurs, it can damage both the overhead wire and the pantograph.
- Acceleration and Rotation of the Pantograph Head: These measures can indicate an incorrectly positioned OCL.
- Contact Forces on the Pantograph: Contact forces can be measured both horizontally and vertically. Deviations in these measures can indicate hard spots and other anomalies in the OCL.
- Detection of Contact Losses: Contact losses can occur due to various reasons and result in electric arcing that accelerates the wear of the carbon strips and of the contact wire.

### 2.5. Requirements from the CRC (Requirements in China)

The OCL monitoring parameters deemed critical by the China Railway Corporation (CRC) are detailed as follows:

- High-Frequency Acceleration: This parameter effectively describes the smoothness of the contact wire, which is relevant to service life, health status, and safety. This is a feasible measurement parameter compared to the high cost of the contact force measurement systems, and it remains important regardless of the current collection quality.
- Rotation of Gear Wheel: Monitoring the rotation of the catenary’s tensioning wheel can provide information on tension variations due to climate change and other dynamic performances.
- Steady-Arm Inclination: Monitoring the inclination of the steady arm can provide insights into the geometrical distortion of the catenary in long-term operation. This measurement can be used as an indicator to support effective maintenance strategies.
- Catenary Cantilever Imagery: Using a low-speed train to capture clear photos of the catenary cantilever system, for example, at night, can prove beneficial. Advanced image processing methods can then be used to automatically detect faults, providing an alternative to conventional visual inspection.
- Steady-Arm Uplift: The uplift of the steady arm should be monitored as it needs to be restricted within a certain safety range. This monitoring can be real time if the power supply and robustness of the system are properly addressed.
- Contact Point Position: Real-time monitoring of the contact point is a crucial indicator to evaluate the risk of dewirement. Advanced image processing methods are needed to automatically track the contact point during operation.

## 3. Data Acquisition Systems

**Figure 3:**
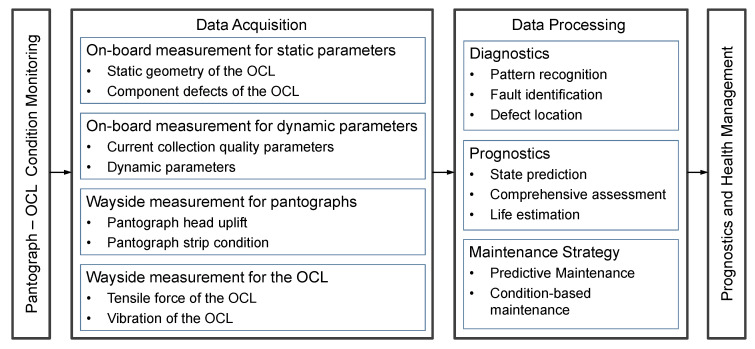
Schematic of pantograph–OCL condition monitoring.

Generally, there are four types of measurement systems to acquire the data from current collection assets: On-board Measurement of Static parameters (OMS); On-board Measurement of Dynamic parameters (OMD); Wayside Measurement of Pantograph (WMP); and Wayside Measurement of OCL (WMO). A schematic representation of the pantograph–OCL condition monitoring is shown in Figure 3.

The data collected by the sensing systems need to be processed in order to be transformed into useful information that can be used to support decision-making and asset management. The data processing typically has three stages:

- The Diagnostics Phase: Pattern recognition is used to identify signatures of defects and locate the faults in the pantograph–OCL system.
- The Prognostics Phase: Long-term measurement datasets are used for comprehensive assessment and to predict the degradation trends of the pantograph–OCL components.
- Condition-Based or Predictive Maintenance Phase: In line with the diagnostic and prognostic results, strategies are developed to improve maintenance efficiency and reduce life-cycle costs of the assets.

### 3.1. On-Board Measurement of Static Parameters (OMS)

#### 3.1.1. Measurement Method

The OMS usually run an inspection vehicle or trolley equipped with sensors and cameras to acquire static geometric parameters and detect defective components of the OCL. The catenary monitoring includes contact wire height, contact line thickness, stagger, etc. These parameters are crucial indicators for evaluating the safety and health condition of the OCL, which may have a noticeable effect on the quality of current collection. Defective support devices are mainly related to loose, cracked, fractured, broken, or even missing components (i.e., the steady arm, insulator, claw, clevis, and double tube joint). The OMS also detect foreign objects on the OCL, e.g., bird nests, plastic bags, and rocks clipped in the insulators.

The detection of the contact wire height, stagger, and contact line wear mainly relies on non-contact detection based on image processing techniques [46,47]. In general, the detection system consists of laser emission, image acquisition, and image processing tools installed on the inspection vehicle. The laser emitter shoots a light towards the contact line, generating a bright spot. The cameras capture the bright spot and transmit the image to the processing module. Usually, the wheelset motion of the inspection vehicle is monitored as well to compensate for the measurements and reduce the noise caused by the vehicle vibration.

There are also camera-based monitoring systems to detect defective OCL components, which are usually mounted on the roof of a railway vehicle. To avoid the interference of complex backgrounds, the inspection is generally performed during the night. The cameras continuously monitor the OCL support devices in global and local views from both the front and reverse sides. The location data, such as the pole number and mileage mark, are recorded using GPS (the Global Positioning System). The component defects are identified by image processing techniques, including pattern learning [48] and deep learning [49].

#### 3.1.2. Application Examples

Several application examples of OMS are presented in Table 3, which demonstrate the developmental stage of the technologies. In the table, a ‘1’ indicates quantities/parameters that can be measured, while ‘0’ represents parameters that the system is not able to monitor [50].

#### 3.1.3. Current Issues and Perspectives

Current issues and perspectives associated with on-board monitoring for static parameters include the following:

- Regarding static geometry detection, the results may be contaminated by the vibration of the inspection vehicle. An important technical issue is to develop high-precision compensation techniques for such vibrations.
- Regarding the catenary component detection, small catenary parts, such as isoelectric lines [51], clevis, and pins, are not visible and can be affected by trees and illumination conditions due to the complex environment. The crucial next step is to improve the identification rate by using advanced computer vision algorithms, e.g., artificial intelligence techniques and deep learning neural networks.

### 3.2. On-Board Measurement of Dynamic Parameters (OMD)

#### 3.2.1. Measurement Method

The OMD systems are usually installed on inspection vehicles or passenger trains in order to acquire dynamic parameters to assess the pantograph–OCL current collection quality. The dynamic parameters include the contact wire uplift, pantograph head acceleration, dynamic stagger, and hard spots. The current collection quality parameters comprise mainly the contact forces and arcing. These parameters are the crucial indicators for the operation of the pantograph–OCL system.

**Figure 4:**
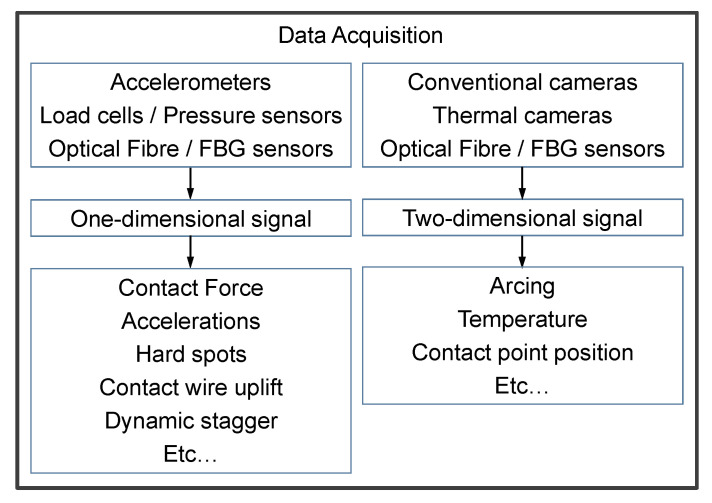
Overview of on-board measurement methods for dynamic parameters.

An overview of the OMD methods is shown in Figure 4. The detection of the accelerations, contact forces, and contact wire uplift mainly relies on the accelerometers, load cells, and Fibre Bragg Grating (FBG) sensors installed on the pantograph. The detection of arcing and the contact point position is mainly achieved by image processing techniques of conventional or thermal images. The following two subsections describe the measurement methods of contact forces and arcing.

#### 3.2.2. Measurement of Contact Forces

Measurement techniques for contact forces have experienced long-term evolution, from the traditional sensors such as accelerometers to optical fibre sensors, including advanced non-contact measurement methods. The three main techniques are described as follows.

The traditional method to monitor contact forces uses accelerometers and load cells. In general, the force sensors are placed on the springs between the contact strips and the pantograph collector [52]. The accelerometers are located on the bottom of the collector strips. According to EN50317 [53], the contact force should be calculated by considering the force measured on the load cells, corrected by the inertial effect of the contact strips and by the influence of the aerodynamic forces.

An alternative method to calculate pantograph–OCL contact forces is by using optical fibre technology. FBG optical strain gauge sensors are embedded into the inner surface of the strip [54], and reference temperature sensors are used for temperature compensation, usually mounted near to the strain sensors [55]. The inertial force is measured by accelerometers that are mounted diagonally on the pantograph head. The most significant advantage of this technique is that the data collected from the FBG sensors is not distorted by the electromagnetic interference arising from the high-voltage environment.

The third methodology to measure contact forces proposes an idea like the above-mentioned established in EN50317, but only the spring reaction forces and the inertia forces have to be measured to obtain the contact forces [56]. The spring reaction forces are obtained by the product of the spring coefficient by the spring deformation, which corresponds to the relative displacement between the contact strip and the pantograph head. The inertia force can be obtained by the product of the accelerations, which can be obtained as the second derivative of the displacements of the contact strip, and the corresponding equivalent mass of the collector strips. In this technique, markers are put on the contact strips and on the pantograph head as targets for the image processing algorithm. Cameras are then mounted on the roof of the train near the pantograph, together with proper lighting devices, to take clear images of the markers. The displacements are obtained by using image processing technology with pattern matching based on the Normalised Cross-Correlation (NCC) method [57].

#### 3.2.3. Measurement of Arcing

Currently, image processing-based methods are widely utilised for arcing monitoring of the pantograph–catenary system; however, the images are very easily affected by environmental conditions such as trees and illumination. Thermal imaging is preferred because it is not affected by environmental issues. This technique is widely used in the railway industry to detect arcing. However, thermal imaging detection techniques can be expensive and challenging to implement in real time due to the amount of data to be processed.

A less expensive method, when compared with the use of thermal cameras, is a photosensitive device (photodiode), which gives a continuous output signal that is related to the presence of an electric arc. Another measurement method to detect the arcing is to examine the current and voltage signals that arrive at the train. A brief introduction to these techniques is given in the following.

Conventional or thermal cameras can be used on the roof of trains to measure arcing [50]. The most utilised technique employs edge detection and Hough transform [58] to locate the position of the pantograph. The position of the contact wire can also be obtained using normal/thermal images [59].

An alternative methodology consists of using photosensitive devices for arcing monitoring. The system uses photomultiplier tubes to measure the duration of the ultraviolet emission due to electric arcing [60]. The major advantages of this system are associated with the fact that it is non-invasive with respect to the pantograph equipment, it is reliable, and it is low cost. Correlation with data acquired during high-speed test runs from different sensors, e.g., currents measured in an equipotential wire between the front and the rear pantographs or images acquired from a TV camera, provides the opportunity to perform an efficient calibration and analysis of selectivity of the phototube sensors.

The third methodology to measure arcing is based on the analysis of the current and voltage signals. In particular, SNCF (Société Nationale des Chemins de Fer) in France measure arcing through monitoring the voltage and electric current in the train engines. This technique assesses the pantograph–catenary interaction performance using the arcing information.

#### 3.2.4. Application Examples

Several application examples of OMD are presented in Table 4 and Table 5, illustrating the stage of development of the technologies. As previously mentioned, ‘1’ is used to indicate quantities/parameters that the sensing system is able to measure, and ‘0’ indicates parameters that the system is not able to monitor.

#### 3.2.5. Current Issues and Perspectives

Current issues and perspectives associated with on-board monitoring for dynamic parameters include the following:

- Inspection Vehicles: The current measurement of key pantograph–catenary dynamic parameters, e.g., contact forces and accelerations, is generally performed using special inspection vehicles. This only gives indications for standard periodic maintenance operations but cannot monitor short-term degradation or defects that may disrupt regular service. Real-time condition monitoring is highly desirable, with a tendency for more train fleets, which operate at commercial speeds, to have monitoring systems installed on board. Reliability and versatility of the measurement systems need to be improved to ensure efficient performance.
- Accuracy of Contact Force Measurement: Traditional sensors are susceptible to distortion by the electromagnetic environment. The temporary fitting of instrumentation may also affect the current collection quality and skew results at the testing stage.
- Assessment Indicators: Due to the difficulties existing in the measurement of contact forces, some researchers propose to use the accelerations to identify the behaviour of the contact forces. The statistics of the acceleration RMS value can be used to detect defects on the contact line and hard spots [61]. The advantage of this technique is that only two accelerometers are installed on the collector strip to capture the accelerations of the pantograph head, and the frequency can be extended up to 200 Hz. Such data can adequately describe the high-frequency characteristics of the flexible registration strips and the irregularities of the contact line.
- Arcing Measurement: Normal imaging techniques are easily affected by environmental and lighting conditions. Thermal imaging is a promising technique for detecting arcing. However, this technology can be expensive and challenging to implement in real time because of the considerable amount of data to be processed. Compared to thermal cameras, photosensitive devices are less expensive, giving a continuous signal output that is related to the presence of an electric arc. Another promising measurement method is to examine the current and voltage input signals that arrive at the train. The reliability of the arcing measurement for the assessment of current collection performance is still not clear, and it does not allow for measuring important safety thresholds such as contact wire uplifts and maximum forces.

### 3.3. Wayside Measurement of Pantographs (WMP)

#### 3.3.1. Measurement Method

The WMP systems are usually camera-based devices installed on wayside poles or beams to monitor the pantographs passing through. Examples of pantograph dynamic parameters measured include the pantograph head uplift and the pantograph strip conditions, such as wear levels, chipping, burning, and cracking.

#### 3.3.2. Application Examples

Several application examples of WMP are presented in Table 6, revealing the developmental stage of these technologies. In this table, ‘1’ indicates parameters that can be measured, and ‘0’ indicates the ones that the system is not able to monitor [50,62].

#### 3.3.3. Current Issues and Perspectives

Current issues and perspectives associated with wayside monitoring for pantographs include the following:

- The high-resolution cameras are expensive, which causes an increase in the cost of the measurement system.
- The image quality is restricted by the distance between the pantograph and the measurement equipment. Devices installed further away make it difficult to capture clear images, whereas a closer installation of equipment could introduce some safety issues.
- The measurement device should work during the day and night and under different weather conditions. The detection algorithm should have high robustness to all these conditions.
- Different styles of the pantograph and of the roof of the train may affect identification. The detection algorithm should be robust enough for all these cases.

### 3.4. Wayside Measurement of the OCL (WMO)

#### 3.4.1. Measurement Method

The WMO uses wayside sensors to monitor the tension and vibration of the catenary assets, including the acceleration and uplift of the wires. The tension device is also used to monitor the tensile forces and the tension wheel rotation in both the contact and messenger wires.

#### 3.4.2. Application Examples

Several application examples of WMO are presented in Table 7, demonstrating the developmental stage of the technologies, where ‘1’ indicates quantities that can be measured, and ‘0’ indicates the ones that cannot be measured [50,63,64,65,66].

#### 3.4.3. Current Issues and Perspectives

Current issues and perspectives associated with wayside monitoring for the OCL include the following:

- The wayside monitoring for catenary vibration is still in the laboratory stage. The interference of the device on the measured results, the electromagnetic effects, and the vibration robustness should be investigated further.
- There is no relevant standard regarding the wayside monitoring for catenary vibration. The selection of indicators and the definition of the threshold values require more research.

## 4. Conclusions and Perspectives

In the context of the current railway industry, developing a real-time condition monitoring programme for diagnostics and prognostics, which is condition-based and achieves predictive maintenance purposes, is a crucial challenge for life-cycle cost reduction and competitiveness, especially when asset management is still based on the age of the assets. By moving towards a condition-based approach, this will allow for optimised asset life and maintenance planning. Designing a Prognostics and Health Management (PHM) solution for the pantograph–OCL system, which is geographically distributed, is not an ordinary task when compared to usual studied systems in the PHM field. To this aim, a comprehensive review of means for pantograph–OCL condition monitoring is conducted here, and several types of data acquisition systems are described. The following provides brief summaries on the current issues and perspectives in industry requirements and data acquisition for the pantograph–OCL system.

### 4.1. Rail Industry Requirements

Requirements of a monitoring system are dependent on end-users’ goals. Understanding what parameters each sector of the rail industry requires is vital for choosing or designing the correct system. In this work, the requirements from several organisations are collected within industry groups, including manufacturers, infrastructure managers, and operators.

From an industry survey carried out by Schunk, the top five functions for each group have been identified, while also listing the key functions as a whole. From this study, it is observed across all sectors that only the dynamic uplift of the contact wires and accident investigation are important to all end users. Parameters such as OCL geometry and a service dashboard are only considered important to individual sectors and their needs. This is also seen from the information given by Network Rail, ProRail, and CRC, who are all infrastructure managers. For them, features such carbon strip wear measurement and service dashboards are not required as this does not directly affect their operation in the same way carbon strip wear would for operators or equipment manufacturers.

In conclusion, the information obtained in this work identifies that each industry group is required to measure and output different parameters with some crossover between them. Designing and developing a system to perform all this for each end user is not feasible. Instead, a network of systems needs to be used to help draw a picture of the infrastructure/OCL and rolling stock/pantograph conditions, where data can be passed among the different interested stakeholders.

### 4.2. Data Acquisition Systems

The detection technique of the OCL static parameters is relatively developed, but the compensation of measurement carrier (inspection vehicle) vibration remains to be an important issue as it can contaminate the measurement results.

For the pantograph–catenary dynamic parameters, the critical measurement items are the contact forces and arcing, which directly reflect the current collection quality. The traditional measurement of contact forces relies on load cells, accelerometers, and strain sensors installed on the collector strips; however, these techniques are complex and have interference in the measurement results and the aerodynamics of the pantograph. The non-contact monitoring methodologies seem to be promising for tackling these issues, however, the robustness of such methods requires further evaluation.

For the detection of arcing, the conventional camera-based systems are very easily affected by obstacles in its complex working environment and by the lighting conditions. Even though thermal imaging is not affected by environmental conditions, this technique is more expensive and is challenging to implement in real time because of the large amount of data to be processed. Another two methods to measure the arcing are photodiodes and the train input current monitoring. These two methods are both economical and easy to deploy; however, the identification rates of these two methods require further investigation.

The wayside monitoring for pantograph equipment has been deployed in most networks all over the world. The biggest issue is the installation position of the cameras, which needs to be close for the system to capture clear images of the pantograph strip, but this can challenge the safe operation of the railway service.

The wayside monitoring of the OCL equipment is an essential part of modern smart railway architecture, which deploys sensors on the OCL to monitor the health conditions of the critical components. Most of these types of measurements are still in the experimental stage. The interference of the device to the measured results, the electromagnetic fields, and the vibration should be investigated further. There is no relevant standard regarding this type of measurement systems. The selection of indicators and the definition of the thresholds require more research as well.

## Figures and Tables

**Table 1:** Train delays due to OCL incidents.

	2019–2020			2020–2021		
Railway Period	No. of Incidents	Trains Delayed	Delay Minutes	No. of Incidents	Trains Delayed	Delay Minutes
1	3	2159	11,885	3	466	4959
2	3	3901	29,223	4	1499	11,254
3	6	3846	26,050	6	1900	12,536
4	2	1387	9885	1	315	1336
5	11	11,154	87,045	9	3393	31,127
6	0	0	0	1	689	5683
7	0	0	0	1	319	2297
8	3	6633	44,569	5	2317	23,132
9	2	2934	22,611	4	2156	12,957
10	1	362	2016	3	883	10,245
11	3	1923	14,724	3	999	5892
12	4	3925	28,567	3	1463	10,860
13	1	3763	21,654	3	765	4336

**Table 2:** Assessment criteria according to EN50367:2020.

Criterion	Threshold
Mean Contact Force (F_m_)	F_m_ = 0.00097 v^2^ + 70 N
Standard Deviation (S_max_)	S_max_ < 0.3 F_m_
Maximum Contact Force (F_max_)	F_max_ < 350 N
Maximum CW Uplift of Steady-arm (D_UP_)	D_UP_ ≤ 120 mm (indicative value)
Percentage of Real Arching (NQ)	NQ ≤ 0.2%

**Table 3:** Capabilities of the on-board measurement systems for static parameters.

Technology	Contact Forces	CW Uplift	Arcing	Strip Wear	Hard Spots	Contact Position	OCL Geometry	CW Wear	Report to Train	Imagery	Trash in OCL
High-Precision Catenary-Checking Monitor System (CRC)	0	0	0	0	1	0	1	1	0	1	1
Selectra Vision CAT-VW	0	0	0	1	1	0	1	1	0	1	0
Selectra Vision CAT-LW	0	0	0	0	0	1	1	1	0	1	0
MERMEC Longitudinal Defects Detection System	0	0	0	0	1	1	1	1	1	0	0
MEIDENSHA Conventional Commercial Service Car	0	0	0	1	1	1	1	1	1	1	1
MEIDENSHA Maintenance Vehicle/Road–Rail Vehicle	0	0	0	1	1	1	1	1	1	0	1
D. Wehrhahn: Online Contact Line Measuring System	0	1	0	0	0	1	1	0	0	0	0
Century: Handheld Intelligent Catenary System	0	0	0	0	1	0	1	1	0	1	0
Riegl: Mobile Mapping System	0	0	0	0	0	0	1	0	0	1	0
DMA: Catenary Monitoring	0	0	0	0	0	1	0	1	0	1	0

**Table 4:** Capabilities of the on-board measurement systems for dynamic parameters, A.

Technology	Contact Forces	CW Uplift	Arcing	Strip Wear	Hard Spots	Contact Position	OCL Geometry	CW Wear	Report to Train	Imagery	Trash in OCL
Comprehensive Pantograph and Catenary Monitor System (CRC)	1	1	1	0	1	1	0	0	0	0	0
Catenary Checking Video Monitor System (CRC)	0	0	1	0	0	0	0	0	0	1	1
Ricardo CatMon	0	0	0	0	1	0	0	0	1	0	0
Transmission PANDAS	0	0	0	0	1	0	0	0	1	1	0
MEIDENSHA Corporation	1	1	1	0	1	0	0	0	0	0	0
Serco Overhead Line Monitoring Systems	0	0	0	0	1	0	0	0	1	1	0
Serco Attended Pantograph Monitoring	1	1	1	0	1	1	0	0	1	1	0
D-RAIL Technical Solution	1	1	1	1	1	0	0	1	1	1	1
DTI: Pantograph and OHL Inspection System	1	0	0	1	0	0	0	0	1	1	0
CONTACT: Pantograph Control System	1	1	0	1	1	0	0	1	0	0	0
SenTech Analytics: IoT Pantographs	1	0	0	0	1	1	0	0	1	1	0

**Table 5:** Capabilities of the on-board measurement systems for dynamic parameters, B.

Technology	Contact Forces	CW Uplift	Arcing	Strip Wear	Hard Spots	Contact Position	OCL Geometry	CW Wear	Report to Train	Imagery	Trash in OCL
Sengenia: Fibre optic sensing	1	0	0	0	1	1	0	0	1	1	0
HBK railway pantograph overhead line monitoring	1	1	0	1	1	1	0	1	0	0	0
MERMEC: Pantograph–catenary interaction measurement	1	1	0	0	1	0	0	0	0	0	0
Deutzer: Position–shock system	0	1	0	0	1	1	0	0	1	1	0
DraadData: Contact wire inspection	1	1	0	1	1	0	0	1	0	0	0
DMA—Wire geometry + wear	1	1	0	1	1	1	0	1	0	0	0
Pantohealth: Pantograph monitoring and diagnostic system	1	1	1	1	1	0	0	0	1	1	1
OLErt: Overhead line and pantograph monitoring	1	1	1	0	1	1	1	0	1	1	0
Balfour Beatty: Attended TrueOHL™	0	1	1	0	0	1	0	1	1	1	0
Balfour Beatty: Unattended TrueOHL™	0	1	1	0	0	1	0	0	1	1	0
Balfour Beatty: Unattended TruePan™	1	0	1	0	1	1	0	0	0	0	0

**Table 6:** Capabilities of the wayside measurement systems for pantographs.

Technology	Contact Forces	CW Uplift	Arcing	Strip Wear	Hard Spots	Contact Position	OCL Defects	CW Wear	Report to Train	Imagery	Trash in OCL
Catenary and Pantograph Video Monitoring System (CRC)	0	0	0	1	0	0	1	0	1	0	0
CAMLIN Group: PANTOBOT 3D	0	0	0	1	0	0	1	0	1	1	0
Ricardo Rail PanMon	0	1	0	1	0	0	1	1	0	1	0
MEIDENSHA Pantograph Monitoring System	0	0	0	1	0	0	1	0	0	0	0
Duostech Automated Pantograph Inspection System	0	0	0	0	0	0	1	0	0	1	0
Image House: PANTOINSPECT	0	1	0	1	0	0	1	0	0	1	0
Selectra: PantoCheck	0	1	1	1	0	0	1	0	0	1	0
DMA: Pantograph Uplift System	0	1	0	1	0	0	1	0	0	1	0
Acoustic Diagnostic of Pantograph Current Collector	0	0	0	1	0	0	0	0	0	0	0
CRRC Dynamic Detection System for Pantograph	1	0	0	1	1	1	0	1	1	0	1

**Table 7:** Capabilities of the wayside measurement systems for the OCL.

Technology	Contact Force/ Position	CW Uplift	Arcing	Hard Spots	OCL Wear/ Geometry/ Defects	Report to Train	Imagery	Trash in OCL	Wire Tension	Temps
Ground Monitor System for Catenary and Power Supply (CRC)	0	1	0	0	0	0	0	0	1	0
SIEMENS Sicat CMS	0	1	0	0	0	0	0	0	1	0
Railway Catenary Structure Monitoring System (NTNU)	0	1	0	0	0	0	0	0	0	0
High-Speed Catenary Non-Contact Monitoring System (SWJTU)	0	1	0	0	0	0	1	0	0	0
Serco Attended/Unattended Trackside Measurement of Dynamic Wire Uplift	0	1	0	0	0	0	0	0	0	0
KRUCH: Contact Wire Uplift Sensor	0	1	0	1	0	0	0	1	1	0
KRUCH: Contact Wire Temperature Sensor	0	0	0	0	0	0	0	0	0	1

## Data Availability

The original contributions presented in this study are included in the article. Further inquiries can be directed to the corresponding author.

## Abbreviations

The following abbreviations are used in this manuscript:

ADD	Automatic Dropping Device
CM	Condition monitoring
CRC	China Railway Corporation
CW	Contact wire
FBG	Fibre Bragg Grating
GPS	The Global Position System
MW	Messenger wire
NCC	Normalised Cross-Correlation
OCL	Overhead contact line
OEM	Original equipment manufacturer
OMD	On-board Measurement of Dynamic parameters
OMS	On-board Measurement of Static parameters
PHM	Prognostics and Health Management
WMO	Wayside Measurement of OCL
WMP	Wayside Measurement of Pantographs
